# Diagnostic accuracy of a new fluoroenzyme immunoassay for the detection of TSH receptor autoantibodies in Graves’ disease

**DOI:** 10.1007/s13317-018-0102-4

**Published:** 2018-02-12

**Authors:** Danilo Villalta, Federica D’Aurizio, Mirella Da Re, Debora Ricci, Francesco Latrofa, Renato Tozzoli

**Affiliations:** 1 Immunology and Allergy Unit, S. Maria degli Angeli Hospital, Pordenone, Italy; 2 Clinical Pathology Laboratory, University Hospital, Udine, Italy; 3 Department of Clinical and Experimental Medicine, University of Pisa, Pisa, Italy; 4 Clinical Pathology Laboratory, S. Maria degli Angeli Hospital, Pordenone, Italy

**Keywords:** Thyrotropin receptor autoantibodies (TRAbs), Graves’ disease (GD), Immunometric assays, ELiA^™^ anti-TSH-R assay

## Abstract

**Purpose:**

Thyrotropin receptor (TSHR) autoantibodies (TRAbs) are a hallmark of Graves’ disease (GD). The aim of this study was to evaluate the diagnostic accuracy of a new third generation automatic fluorescence enzyme immunoassay for TRAb measurement in GD, in comparison with two current IMAs.

**Methods:**

Sera of 439 subjects (57 patients with untreated GD, 34 with treated GD, 15 with GD and Graves’ orbitopathy, 52 with multinodular non-toxic goiter, 86 with Hashimoto’s thyroiditis, 20 with toxic adenoma or toxic multinodular goiter, 55 with non-thyroid autoimmune diseases and 120 normal controls) were tested for TRAbs with the ELiA^™^ anti-TSH-R assay (ThermoFischer Scientific, Uppsala, Sweden), the TRAK^™^ RIA, Brahms (Thermo Scientific, Hennigsdorf, Germany) and the Immulite^™^ TSI assay (Siemens Healthcare, Llanberis, UK).

**Results:**

Sensitivity and specificity of the ELiA™ anti-TSH-R assay, TRAK™ RIA and Immulite™ TSI assay were 94.7% and 99.6, 100 and 98.2%, 100 and 98.2%, respectively. Spearman’s coefficient and Passing-Bablok regression showed a satisfactory correlation between EliA™ and TRAK™ [rho: 0.925; 95% CI: 0.883-0-953. Intercept: − 0.875 (95% CI: − 2.411 to 0.194); slope: 1.086 (95% CI: 0.941 to 1.248)], and between ELiA^™^ and TSI^™^ [rho: 0.947; 95% CI: 0.912 0.969. intercept: 1.085 (95% CI: 0.665 to 2.116); slope 1.315 (95% CI:1.116 to 1.700)].

**Conclusions:**

The diagnostic performance of ELiA^™^-TSH-R assay is comparable to that of some current TRAb assays. It may be adopted into clinical practice for the differential diagnosis of hyperthyroidism, to screen for transient hyperthyroidism, and to monitor disease activity and treatment effects.

## Introduction

The thyrotropin receptor (TSHR), expressed on the cell surface of thyrocytes, initiates the major signals that direct thyroid cell growth and hormone synthesis/secretion [1]. In addition, it is now well-established that TSHR is expressed in a variety of extra-thyroidal cells, including fibroblasts, adipocytes and bone cells, where it is known to modulate target cell function [2, 3]. The TSHR is a target autoantigen in Graves’ disease (GD) [4–6], where TSHR autoantibodies (TRAbs) induce thyroid growth and hyperthyroidism and represent an important diagnostic hallmark. TRAbs are also detected in a small portion of patients with Hashimoto’s thyroiditis (HT) [5].

Three varieties of TRAb are recognized: stimulating (S-TRAbs), blocking (B-TRAbs), and “neutral” (neutral TRAbs) autoantibodies. Some authors report that S-TRAbs preferentially recognize the N-terminal region, while B-TRAbs are more biased toward the C-terminal region of the ectodomain of the TSHR [7, 8]. Neutral TRAbs are reported to be directed against the cleavage region of the TSHR and are able to induce apoptosis in thyrocytes [9]. However, experimental evidence, including the analysis of the crystal structure of the TSHR extracellular domain bound to stimulating or blocking human monoclonal autoantibodies, show that TRAbs bind extensively across the leucine rich repeats of the TSHR extracellular domain, regardless of biological activity [10]. TRAbs without stimulating activity can be enriched from normal individuals [11].

In the last 50 years, bioassay (BA) and immunoassay (IMA) methods have been used to detect autoantibodies against the TSHR. BAs measure functional activity of TRAbs, (S-TRAbs, B-TRAbs), while IMAs measure the binding of autoantibodies to the receptor (total TRAbs, T-TRAbs) and are not able to differentiate S-TRAbs from B-TRAbs [12]. Since S-TRAbs are highly correlated with GD activity, BAs result as the optimal method for TRAb detection in GD. They are, however, cumbersome, time-consuming and in need of optimization and standardization [13], thus it remains restricted to a limited number of specialized laboratories. On the contrary, second and third generation IMAs are suitable for clinical practice and show high analytical and clinical accuracy [14, 15], even if they do not allow to distinguish between the different kinds of TRAbs found in patients with AITDs. However, recently, an assay using chimeric TSHR putatively detecting S-TRAbs, based on the putative structure of the extracellular domain of the TSHR and its interaction with TSHR antibodies [1], has been developed [16].

The aim of this study was to evaluate the diagnostic accuracy of a new third generation automatic fluorescence enzyme immunoassay (FEIA) for TRAb measurement in GD, in comparison with the current two IMAs.

## Materials and methods

### Patients

Sera of 439 subjects [57 patients with untreated GD, 34 with treated GD (1–12 months of treatment), 15 with GD and Graves’ orbitopathy (GD/GO), 52 with non-toxic multinodular goiter (NTMG), 86 with HT, 20 with toxic adenoma or toxic multinodular goiter (TA/TMG), 55 with non-thyroid autoimmune diseases (NTAD) (systemic lupus erythematosus, rheumatoid arthritis, autoimmune gastritis, celiac disease), and 120 normal controls (NC)] were evaluated. GD subjects were diagnosed according to the American Thyroid Association-American Association of Clinical Endocrinologists guidelines [17].

Patients affected by HT were selected according to the following criteria: ultrasound hypoechogenicity and if the thyroperoxidase antibodies (TPOAbs) levels were higher than the upper reference limit. NC (60 males and 60 females) were screened during the “Thyroid takes to the square” survey, carried out in the province of Verona (Italy) from 2008 to 2013. These subjects met the NACB criteria: younger than 30 years, TSH between 0.5 and 2.0 mIU/L, normal thyroid ultrasound, absence of autoimmune and non-autoimmune thyroid disease or other autoimmune diseases [18]. All of them gave informed consent for their participation in the study.

### Immunoassays

The ELiA^™^ anti-TSH-R assay is a competitive fully automated fluoroenzyme immunoassay (ThermoFischer Scientific, Uppsala, Sweden). The design of the method is as follows: a human recombinant TSH receptor is immobilized via capture antibody to the well; TRAbs in the patient’s sample (90 μL diluted 1:2 with PBS containing BSA, EDTA, detergent and sodium azide 0.095%) bind to the coated human receptor (sample incubation at 37 °C for 30 min); after washing away unbound components, 90 μL of β-galactosidase-labeled mouse recombinant antibodies (ELiA™ anti-TSH-R Conjugate) are added to form a TSH receptor-conjugate complex with TSH receptors not blocked by serum antibodies. After incubation at 37 °C for 30 min and washing away the unbound conjugate, 90 μL of a development solution (0.01% 4-methylumbelliferyl-β-d-galactoside) is added and the fluorescence activity is measured. The Phadia 250 instrument (ThermoFischer Scientific) automatically processes all steps of the test.

ELiA^™^ anti-TSH-R assay uses a 6-point calibration curve (0–40 IU/L), and is calibrated against the 2nd International Standard (IS) NIBSC 08/204. The cut-off suggested by the manufacturer was 3.3 IU/L.

The results obtained with the ELiA^™^ anti-TSH-R assay were compared with those obtained with the TRAK^™^ Human radio-immunoassay (TRAb RIA, Brahms Termo Scientific, Henningsdorf, Germany), a second generation immunoassay [9], and with the Immulite^™^ TSI assay (Siemens Heathcare, Llanberis, UK).

TRAK^™^ is a manual radio-immunoassay. In the first step, 100 μL of patient’s serum was incubated for 2 h at room temperature in a tube coated with human TSH receptor. After washing, 200 μL of I^125^ labeled TSH was added. Labeled TSH binds to the remaining unoccupied TSH receptors. After incubation for 1 h at room temperature and after the discharge of the free labeled TSH, the tube is counted in a gamma counter. The content of TRAbs in the patient’s sample is inversely proportional to the bound labeled TSH.

Immulite^™^ TSI assay is a fully automated chemiluminescent IMA, designed to detect S-TRAbs. It employs a pair of recombinant human TSHR constructs in a bridging format: the capture and the signal receptor [16]. The capture construct is a TSHR chimera with the N-terminus of human TSHR binding the S-TRAbs, and LH/CG epitope replacing the residues 261–370 putatively binding the B-TRAb. The signal receptor is constructed from a portion of the extracellular domain (aa 21-26) of TSHR. It is fused with secretory alkaline phosphatase (SEAP) in a buffer solution. Briefly, in the first step, 50 μL of patient’s serum is incubated with the solid phase (polystyrene bead) for 30 min, allowing the TRAbs in the sample to bind through one arm of the capture receptor. After washing, the signal receptor is added to the reaction tube and incubated for 30 min. Unbound signal receptor is then removed by centrifugal washes, chemiluminescent substrate is added to the reaction tube and a signal is generated in direct relation to the amount of TRAbs in the sample.

The cut-off levels suggested by the manufactures were 1.5 IU/L and 0.55 IU/L for TRAK and TSI, respectively.

TRAK was calibrated against 1st IS (NIBSC 90/672) and TSI Immulite^™^ against 2nd IS (NIBSC 08/204).

### Statistical evaluation

Statistical analysis was performed by MedCalc software version 10.4.5 (Mariakerke, Belgium) and GraphPad Prism, Version 4.0 (San Diego, CA, USA). A two-sided value of *p* < 0.05 was considered statistically significant.

TRAb values lower than the limit of quantification (LoQ) were considered equal to LoQ for statistical purpose. The normality of TRAb distribution was assessed using the Shapiro–Wilk test for the results of each assay. As serum TRAb values were not normally distributed, TRAb values were described as median with range (minimum–maximum) and the statistical analyses were performed using non-parametric tests, in particular the Kruskal-Wallis test to compare the NC group with the others (GD, GDT, OB, NTMG, NC, AT, NATD).

Receiver operating characteristic (ROC) curve for ELiA™ anti-TSH-R assay was plotted and analyzed to select the best cut-off level. Clinical sensitivity and specificity were also calculated [19].

Correlation, linear association and agreement between assays were assessed by Spearman’s Rank Correlation Coefficient (rho), Passing-Bablok regression analyses and Bland–Altman plots, respectively. The qualitative (positive/negative) agreement between ELiA^™^ anti-TSH-R assay and the other methods was evaluated by means of Cohen’s Kappa.

## Results

**Fig. 1:**
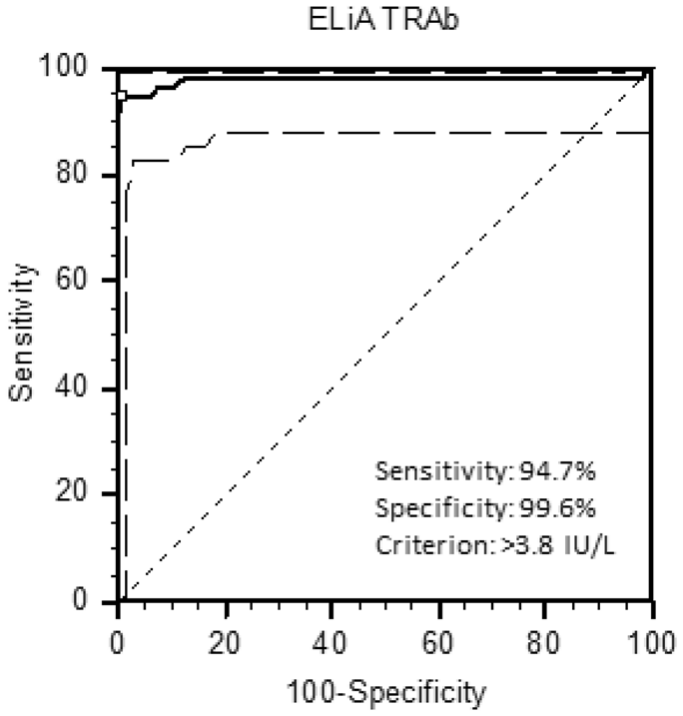
ROC analysis for ELiA^™^ TRAb assay

**Fig. 2:**
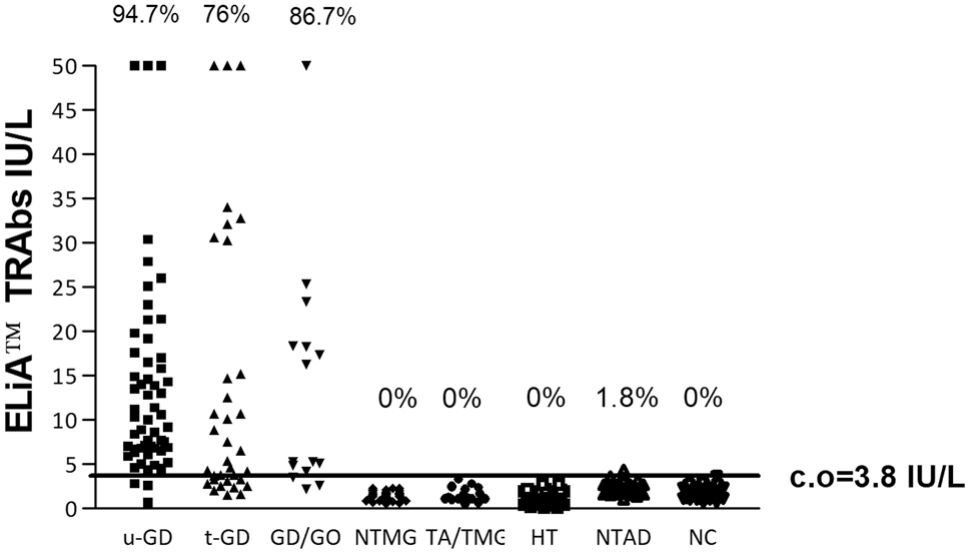
ELiA-TRAb levels in the different groups enrolled in the study. u-GD, Graves’ disease before the treatment, *t-GD* GD on treatment, *GD/GO* Graves’ patients with orbitopathy, *NTMG* multinodular non-toxic goiter, *TA/TMG* toxic adenoma/toxic multinodular goiter, *HT* Hashimoto’s thyroiditis; *NTAD* non-thyroid autoimmune diseases, *NC* normal controls, *%* positive cases, *c.o* cut-off

According to the ROC curve analysis, the optimal threshold to maximize sensitivity and specificity of the ELiA™ anti-TSH-R assay was 3.8 IU/L (Fig. 1), slightly higher than that suggested by the manufacturer. Using this cut-off, the sensitivity for untreated GD was 94.7% and the specificity 99.6%. The percentage of TRAb positivity in all patient groups is shown in Fig. 2, and the median and range (minimum–maximum) values of the TRAbs for each group are reported in Table 1. A statistically significant difference (*p* < 0.0001) was shown between the TRAb levels of NC and those of the GD and GD/GO groups. Values of TRAbs of the treated GD group were significantly lower than those of the untreated GD and GD/GO groups (*p* < 0.01).

**Table 1:** Median and range of the EliA^™^ TRAb values in different groups of patients

Disease	No.	ELiA^™^ TRAb (median IU/L)	Range (minimum–maximum)	Significant difference from NC (p)
u-GD	57	10.0	0.7− > 50	< 0.0001
t-GD	34	5.95	1.6− > 50	< 0.0001
GD/GO	15	16.3	2.2− > 50	< 0.0001
NTMG	52	1.4	0.6–2.3	ns
TA/TMG	20	1.3	1.0–3.3	ns
HT	86	1.1	0.1–2.7	ns
NTAD	55	2.2	1.0–4.4	ns
NC	120	1.4	0.7–3.8	ns
Overall	439			

*u-GD* Graves’ disease before treatment, *t-GD* GD on treatment, *GD/GO* Graves’ patients with orbitopathy, *NTMG* multinodular non-toxic goiter, *TA/TMG* toxic adenoma/toxic multinodular goiter, *HT* Hashimoto’s thyroiditis, *NTAD* non-thyroid autoimmune diseases, *NC* normal controls

Using the TRAK™ and the TSI™ assay, the sensitivity and specificity were 100 and 98.2%, and 100 and 98.2%, respectively. The overall agreement, evaluated using a 2 × 2 classification table, between ELiA^™^ and TRAK^™^ was 97.9% (CI 95%: 96.1–99.0) [positive agreement: 95.3% (CI 95%: 92.2–99.5), negative agreement 98.7% (CI 95%: 97.8–99.5)]; Cohen k: 0.940 (CI 95%: 0.90–0.98). The overall agreement between ELiA™ and TSI™ was 98.5% (CI 95%: 92.0–98.0) [positive agreement: 96.3% (CI 95%: 93.6–99.0), negative agreement: 99.1% (98.4–99.8)]; Cohen k: 0.954 (CI 95%: 0.92–0.98).

**Fig. 3:**
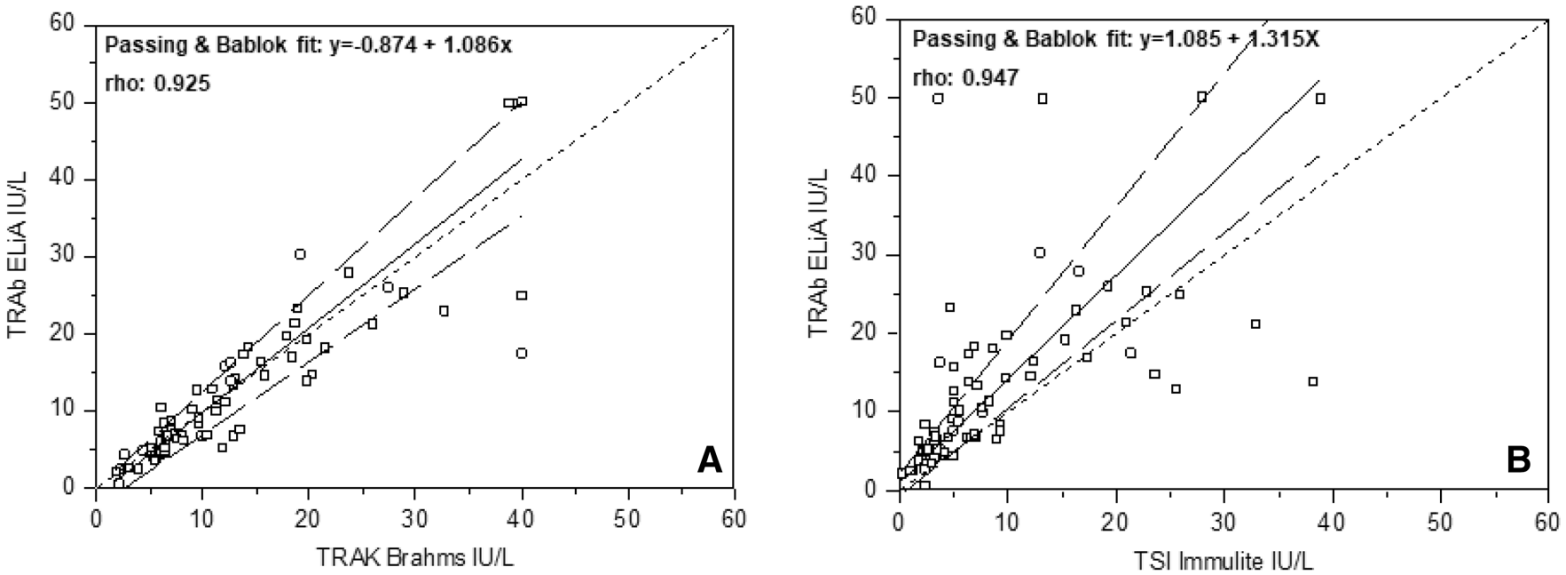
Correlation between ELIA^™^ TRAb assay and TRAK^™^ Assay (**a**), and between ELIA^™^ TRAb assay and TSI^™^ Immulite (**b**) (Passing-Bablok analyses)

Spearman’s coefficient and Passing-Bablok regression showed a satisfactory correlation between EliA^™^ and TRAK^™^ (Fig. 3a) [rho: 0.925; 95% CI: 0.883–0.953. Intercept: − 0.875 (95% CI: − 2.411 to 0.194); slope: 1.086 (95% CI: 0.941 to 1.248)], and between ELiA^™^ and TSI^™^ (Fig. 3b) [rho: 0.947; 95% CI: 0.912 0.969. intercept: 1.085 (95% CI: 0.665 to 2.116); slope 1.315 (95% CI:1.116 to 1.700)].

**Fig. 4:**
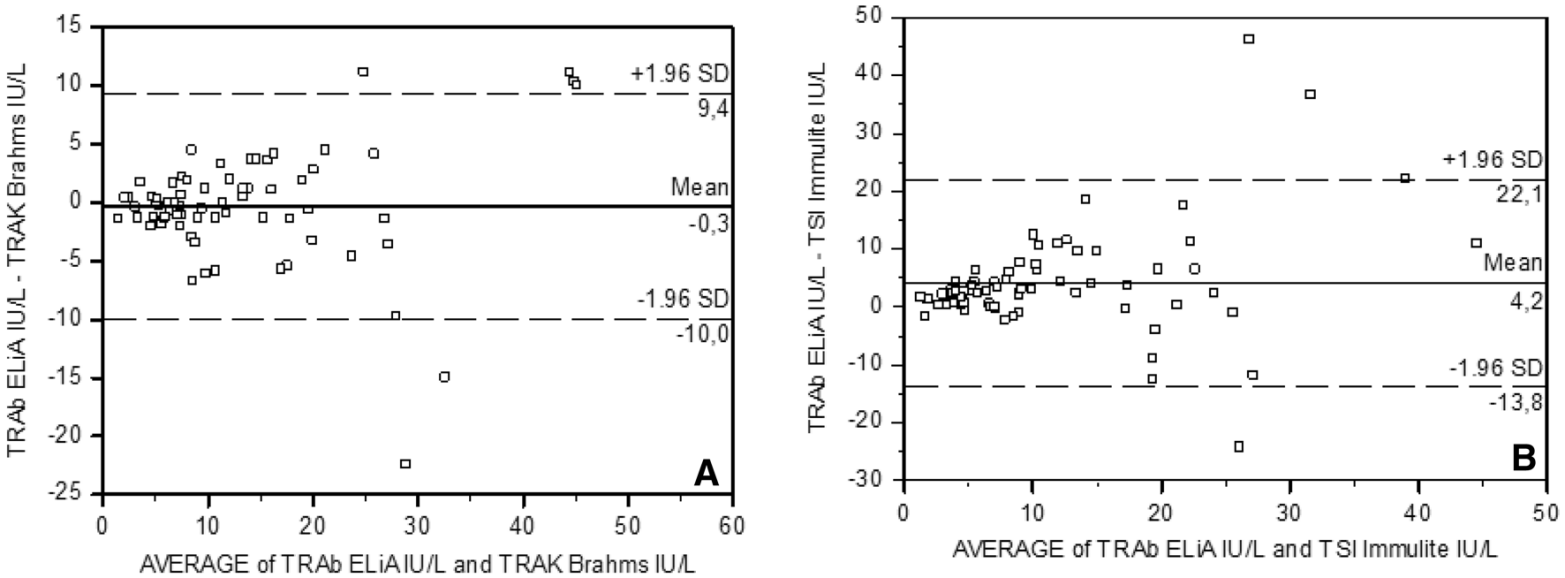
Inter-assay agreement between ELIA^™^ TRAb assay and TRAK™ Assay (**a**), and between ELIA^™^ TRAb assay and TSI™ Immulite (Bland–Altman plots)

Bland–Altman analysis between ELiA™ and TRAK pointed out a bias of − 0.3 IU/L (95% CI: − 10 to +9.4) (Fig. 4a), and between ELiA^™^ and TSI^™^, a bias of 4.2 IU/L (95% CI: − 13.8 to +22.1) (Fig. 4b), showing an acceptable agreement.

## Discussion

TRAb detection is widely accepted as a routine test for diagnosing and monitoring GD and for differential diagnosis of the various forms of hyperthyroidism [20]. In this study, we evaluated the diagnostic accuracy of the new fully automated third generation assay (ELiA^™^-TSH-R assay) for the measurement of TRAbs in comparison with the two current IMAs.

The diagnostic sensitivity of ELiA^™^-TSH-R assay for GD resulted high, though slightly lower than those of the TRAK^™^ and TSI^™^ Immulite assays. In all probability, this is associated to the lower analytical sensitivity of the ELiA^™^-TSH-R assay, as shown by the high cut-off (3.8 IU/L). However, the three patients negative with ELiA^™^-TSH-R assay resulted low positive with the other two assays. On the contrary, the specificity of ELiA^™^-TSH-R assay (99.6%) was slightly higher than those of TRAK^™^ and TSI™ Immulite assays (98.2%). In the control population, only one patient with systemic lupus erythematosus (SLE) showed a low titer of TRAbs (4.4 IU/L). It is not surprising, since SLE is the autoimmune disease associated with the largest number of autoantibodies [21]. Thyroid antibodies, in particular, are frequently associated with this autoimmune disease and are predictive markers of thyroid disorders (hypothyroidism and hyperthyroidism), present in SLE with a high prevalence [22]. No patients with HT showed positivity for TRAbs with the ELiA^™^-TSH-R assay, whereas three cases resulted positive with the other two methods. In the case of TSI^™^ Immulite, putatively measuring only S-TRAbs, this result was unexpected, but confirmed the findings of other studies [15, 22]. The absence of TRAbs in HT with the ELiA^™^-TSH-R assay was also surprising, since previous studies using second and third generation assays detected TRAbs in 5–20% of HT patients [14, 24–26], putatively B-TRAbs or neutral TRAbs. We cannot discriminate whether this represents a higher specificity of the method (i.e., no/low detection of B-TRAbs or neutral TRAbs) or a lower sensitivity, since positive samples obtained with the other two methods were not tested with a BA.

As expected, patients undergoing anti-thyroid drug treatment for 1–12 months showed lower TRAb values, with 24% resulting as negative. The majority of negative patients (7/8) were in clinical remission or euthyroid while on long-term low dose anti-thyroid drugs, confirming the usefulness of TRAb measurement for following disease activity and treatment effects [7, 23].

Correlation and agreement between ELiA^™^-TSH-R assay and the other methods were good, even if it was higher with the TRAK^™^ assay, notwithstanding the latter is calibrated against the first international standard (NIBSC code 90/672). This is likely due to the different designs of the TSI^™^ Immulite assays, as previously described.

In conclusion, the diagnostic performance of the fully automated 3rd generation ELiA^™^-TSH-R assay is at least comparable to that of some current TRAb assays, with a trend toward a higher specificity. As a consequence, it may be adopted into clinical practice for the differential diagnosis of hyperthyroidism (including patients with unusual GD/GO), to screen for transient hyperthyroidism, and to monitor disease activity and treatment effects.
